# Role of Mandibular Parameters in Gender Determination: A Systematic Review and Meta-Analysis

**DOI:** 10.7759/cureus.59965

**Published:** 2024-05-09

**Authors:** Abirami Arthanari, Shanmathy Sureshbabu, Karthikeyan Ramalingam, Monal Yuwanati, Lavanya Prathap, Vignesh Ravindran

**Affiliations:** 1 Department of Forensic Odontology, Saveetha Dental College and Hospitals, Saveetha Institute of Medical and Technical Sciences, Saveetha University, Chennai, IND; 2 Department of Oral Pathology and Microbiology, Saveetha Dental College and Hospitals, Saveetha Institute of Medical and Technical Sciences, Saveetha University, Chennai, IND; 3 Department of Anatomy, Saveetha Medical College and Hospitals, Saveetha Institute of Medical and Technical Sciences, Saveetha University, Chennai, IND; 4 Department of Pedodontics and Preventive Dentistry, Saveetha Dental College and Hospitals, Saveetha Institute of Medical and Technical Sciences, Saveetha University, Chennai, IND

**Keywords:** minimum ramus width, projective ramus height, condylar ramus height, coronoid ramus height, anthropology, meta-analysis, mandibular parameters, mandibular metrics, gender determination, sexual dimorphism

## Abstract

Forensic anthropology and forensic medicine both have been fascinating fields that deal with mandibular characteristics and sex determination. Researchers may determine an individual's biological sex with amazing precision by examining the size, shape, and proportions of the mandible. This information is useful for anthropological studies and criminal investigations. This systematic review aims to evaluate the consistency and validity of using mandibles as a method for gender determination across different populations. A systematic search was conducted in PubMed, Web of Science, and Scopus databases. Further, a manual search was carried out to find additional studies. Mandibular parameters and other relevant data about research were extracted from the included studies. Random effects meta-analysis was carried out for four parameters. A total of nine studies were included in the systematic review out of 76 initial search results. All studies were in vitro. Nine studies were included in the qualitative analysis, whereas only seven studies were included in the meta-analysis. A total of 2385 individuals (1193 male and 1192 female) were evaluated in the included studies. The parameters assessed were as follows: minimum ramus breadth (MiRB), maximum ramus breadth (MaRB), projective ramus height (PRH), bigonial width (BGW), gonial angle (GA), and antegonial angle (AGA). Meta-analysis was conducted for four parameters out of six. For the two parameters, meta-analysis was not conducted as only one study was evaluated. Meta-analyses of PRH obtained a high degree of heterogeneity (99%), mean difference (MD) of 4.06 mm, and p-value of p=0.09. Meta-analysis of BGW obtained 93% heterogeneity, MD of 9.03 mm, and p=0.0007. Meta-analysis of GA showed 99% heterogeneity, MD of 3.44 mm, and p=0.66. Meta-analysis of AGA obtained a low heterogeneity of 30%, MD of -0.77 mm, and p=0.23. The parameter, BGW, can be considered a useful tool in identifying sex. The parameters, PRH, GA, and AGA, cannot be preferred as a reliable tool in identifying the sex of an individual in forensic contexts.

## Introduction and background

Sex determination is a basic component of biological and forensic anthropology and is important in several disciplines, including population genetics, archaeological research, and forensic investigations. It entails determining a person's biological sex from their skeletal remains. The mandible is one of the most important skeletal parts that is frequently analyzed to determine a person's sex because of its sexually dimorphic traits and relatively high preservation rate in forensic and archaeological contexts [1]. The largest and strongest facial bone, the mandible, also referred to as the jawbone, supports the lower face and teeth structurally. The reliability of bone morphology in determining sex lies in the consistency of these traits, which are minimally affected by environmental and genetic factors. The morphological differences between males and females of the same species, known as sexual dimorphism, are most pronounced in the human mandible [2]. Many studies have been conducted on these dimorphic features to create sex estimation techniques. Because there is no soft tissue present, forensic anthropologists frequently find it difficult to identify a person's sexual orientation from skeletal remains. For this reason, skeletal characteristics displaying sexual dimorphism are used by researchers. The analysis of mandibular traits serves as a reliable and non-invasive approach to ascertaining gender, thereby significantly contributing to the resolution of forensic investigations and the reconstruction of population demographics [3].

The size of the mandible is a primary determinant of sex. In general, males possess stronger and larger mandibles compared to females. Sexual dimorphism can be attributed to several factors, such as disparities in biomechanics related to the pressures exerted during biting and chewing, with hormonal influences during the processes of growth and maturity. Anthropologists and forensic experts utilize mandibular length, height, and width measurements to distinguish between male and female individuals [4]. Shape dimorphism, along with size dimorphism, plays a crucial role in distinguishing sex by analyzing mandibular features. Variations in mandibular morphology across sexes are observed in features such as the angle of the mandibular ramus, the form of the mandibular symphysis, and the growth of the mental protuberance. Females typically have a mandibular morphology that is characterized by a rounded and graceful shape, whereas males tend to display more prominent and acute mandibular angles, a bigger mental protuberance, and a more sturdy mandibular body [5]. Advancements in technology have facilitated the examination of mandibular sexual dimorphism through the utilization of three-dimensional imaging techniques such as computed tomography (CT) scanning and geometric morphometrics, alongside traditional morphometric searches. These techniques enhance the accuracy and reliability of sex determination in forensic and anthropological contexts by facilitating a comprehensive analysis of jaw size and shape [6]. In addition, it is also important to consider population-specific variations in mandibular morphology when employing sex prediction methods that rely on mandibular characteristics. Sexual dimorphism exhibits varied degrees across different groups as a result of a confluence of genetic, environmental, and cultural factors. To achieve accurate sex determination based on mandibular traits, it is imperative to establish population-specific criteria and reference databases [7].

The examination of mandibular characteristics to determine sex serves as a valuable illustration of an interdisciplinary methodology that integrates forensic, anthropological, and anatomical techniques [8]. Sophisticated analytical tools and an understanding of the sexual dimorphism inherent in the human mandible enable researchers and forensic specialists to effectively determine an individual's biological sex from skeletal remains. Forensic investigations and the reconstruction of past populations are facilitated by this process [9]. The present systematic review aimed to find evidence about the role of mandibular morphometric parameters in gender determination. 

## Review

Materials and methods

This review was performed under the guidelines of the Preferred Reporting Items for Systematic Reviews and Meta-Analyses (PRISMA). The keywords were elucidated based on PICO (Population, Intervention, Comparison, and Outcome). The research question was defined as "Can mandibular parameters aid in gender determination?".

PICO analysis

The population selected is the human mandible, and the intervention is related to mandibular parameters (coronoid ramus height, condylar ramus height, projective ramus height (PRH), minimum ramus width, gonial angle (GA), ramus flexure, bigonial width (BGW), antegonial depth, antegonial angle (AGA), maximum ramus breadth (MaRB)), which are compared between males and females, and the outcome of the research is gender identification.

Search strategy and sources

The search strategy followed an initial review of the existing literature, employing both free text and controlled vocabulary. A comprehensive electronic search was performed across various databases including PubMed, Web of Science, and Scopus. No specific search limitation was set. To identify possibly eligible publications, gray literature, and specialty journals, a manual search was conducted. Conference papers, letters to editors, and case reports were excluded from the consideration.

Databases searched and manual search

Data search was done using PubMed, Web of Science, and Scopus, and there was no limitation set for publication date and type of publication. The following journals were manually searched to complete the review on the subject of interest: Journal of Forensic Dental Sciences, Journal of Nepal Health Research Council, Journal of Oral and Maxillofacial Pathology, Indian Journal of Dental Research, BioMed Research International, and Journal of Forensic Science Research.

Screening and study selection

Two independent reviewers were involved in searching titles and abstracts to find the relevant articles based on the inclusion and exclusion criteria. Full articles of certainly eligible articles were retrieved to establish the eligibility of the present review. Any disagreements between the two reviewers were resolved by the third reviewer.

Results

**Figure 1:**
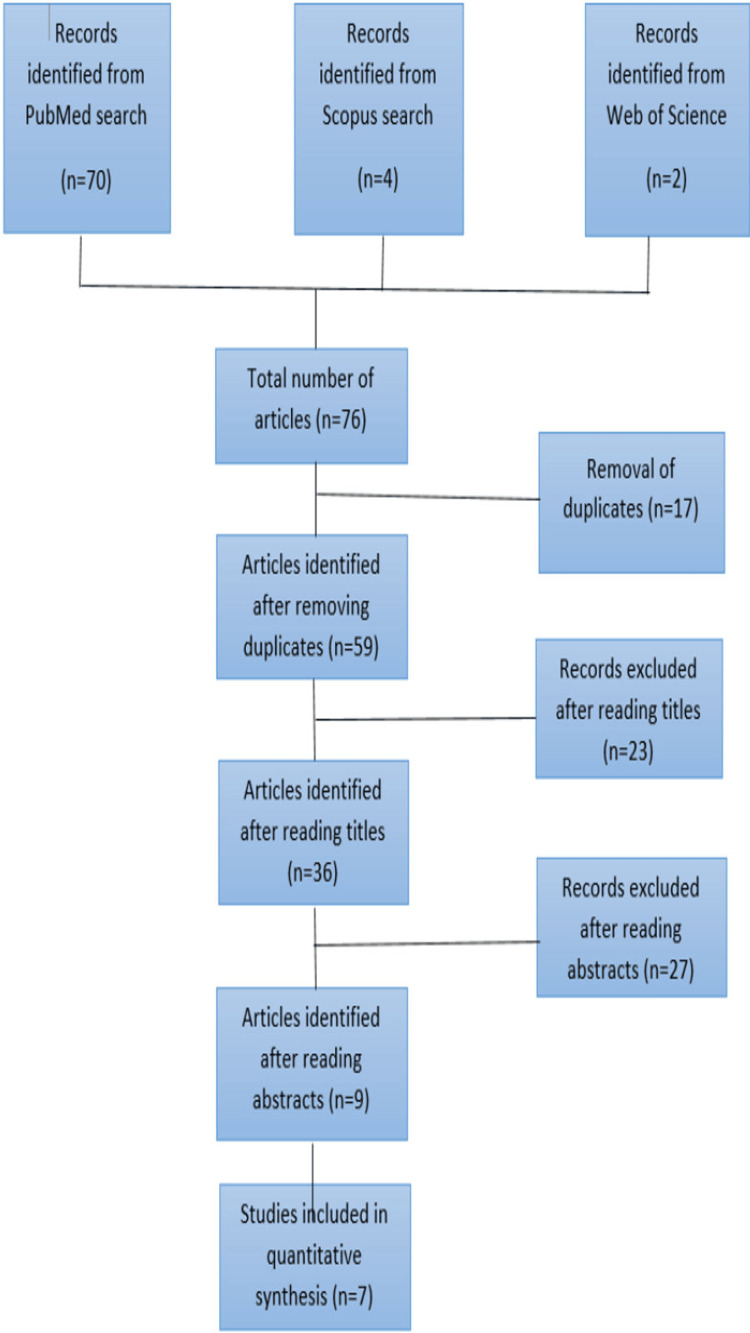
PRISMA flowchart showing the selection of included studies PRISMA: Preferred Reporting Items for Systematic Reviews and Meta-Analyses

A total of 76 articles were obtained from the extensive search. Seventeen articles were excluded considering them as duplicates. After reading the title, 23 articles were excluded. Twenty-seven articles were excluded after reading the abstracts. A total of nine studies were included in the systematic review out of 76 initial search results. All studies were in vitro. Nine studies were included in the qualitative analysis, whereas only seven studies were included in the meta-analysis. A total of 2385 individuals (1193 male and 1192 female) were evaluated in the included studies. The PRISMA flowchart shows the selection of included studies which is mentioned in Figure 1.

Characteristics of the included studies

Among the study characteristics of the included studies, PRH was the most measured parameter (n=3). Other parameters measured were minimum ramus breadth (MiRB) (n=1), MaRB (n=1), BGW (n=2), GA (n=2), and AGA (n=2). The definitions for all these mandibular parameters were given in Table 1 below.

**Table 1:** Definitions for measurements of mandibular parameters PRH: projective ramus height; BGW: bigonial width; GA: gonial angle; AGA: antegonial angle; MiRB: minimum ramus breadth; MaRB: maximum ramus breadth

S. no.	Parameters	Definition
1	PRH	The projective height of the ramus is measured between the highest point of the mandibular condyle and the lower margin of the jaw
2	BGW	BW was measured horizontally between the left and right gonion
3	GA	The GA is measured by taking the tangent to the posterior border of the ramus and the tangent to the lower border of the mandible on the lateral cephalogram
4	AGA	The AGA was measured by tracing two lines parallel to the antegonial region that intersected at the deepest point of the antegonial notch
5	MiRB	The distance between the most anterior point on the mandibular ramus and a line connecting the most posterior point on the condyle and the angle of the jaw
6	MaRB	The smallest anterior-posterior diameter of the ramus

**Figure 2:**
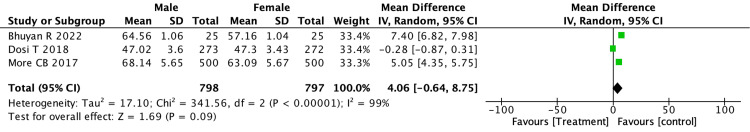
Meta-analysis of PRH in males and females PRH: projective ramus height

All of the studies included two comparative groups: males (n=9) and females (n=9). Outcomes were measured using mean and standard deviation in males and females. In the meta-analysis of PRH in males and females, three studies were included. The meta-analysis of PRH showed a mean difference of 4.06 mm between males and females which was not statistically significant (p=0.09). Therefore, PRH cannot discriminate between males and females which is represented in Figure 2.

**Figure 3:**
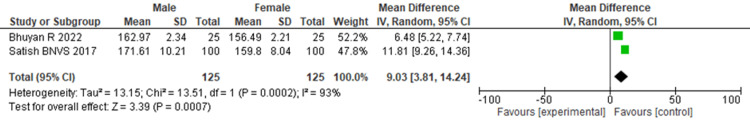
Meta-analysis of BGW in males and females BGW: bigonial width

In the meta-analysis of BGW in males and females, two studies were included. The meta-analysis showed a mean difference of 9.03 mm which showed statistical significance of p=0.0007. Hence, BGW can discriminate between males and females which is represented in Figure 3.

**Figure 4:**
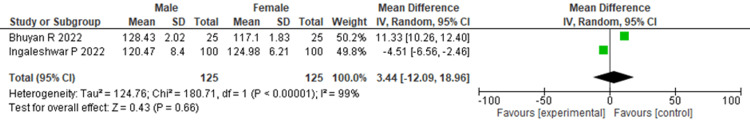
Meta-analysis of GA in males and females GA: gonial angle

In the meta-analysis of GA in males and females, two studies were included. The meta-analysis of the GA obtained a mean difference of 3.44 mm which showed no statistical significance of p=0.66. Thus, GA cannot discriminate between males and females which is represented in Figure 4.

**Figure 5:**
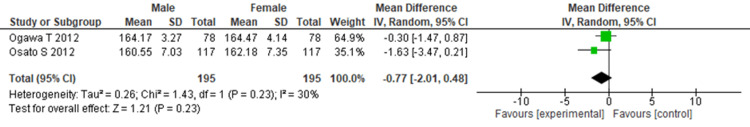
Meta-analysis of AGA in males and females AGA: antegonial angle

In the meta-analysis of AGA in males and females, two studies were included. A low heterogeneity of 30% was observed. The meta-analysis of the AGA obtained a mean difference of -0.77 mm which showed no statistical significance (p=0.23). Therefore, AGA cannot discriminate between males and females which is represented in Figure 5.

Discussion

The results of the systematic review indicate that the parameters, PRH, GA, and AGA, do not show discrimination between males and females, whereas BGW shows significant differences between males and females. This proves that, out of all the parameters assessed, BGW can be a useful tool to assess the gender of an unknown individual.

Mandibular parameters are a method of determining an individual's biological sex by examining certain features of the mandible. This technique makes use of several morphological characteristics of the mandible, including GA, ramus height, and mandibular body measurements [10]. Through the use of advanced methods like digital imaging and geometric morphometrics, researchers can identify minute differences between the mandibles of men and women [11]. This method is important for forensic anthropology, archaeology, and medical diagnosis since it can be used to identify human remains or analyze population patterns. Mandibular characteristics allow for accurate sex determination that benefits many different domains and has many uses in forensic and scientific research [12]. The present study included seven studies after excluding irrelevant articles based on the title and abstracts. 

PRH is the vertical distance between the lowest point of the mandibular notch and the highest point on the mandibular ramus. Because of the sexual dimorphism seen in human mandibles, this value is important. In general, males have higher PRH than females. These variations result in different patterns of growth and development in the mandibles of males and females due to hormonal and genetic effects [13]. Greater muscular attachment regions and higher biomechanical stress are reflected in the larger and more robust mandibular dimensions found in males with increased PRH. To ascertain the biological sex of skeletal remains, anthropologists and forensic professionals frequently use PRH measures in combination with other mandibular characteristics [14]. For precise sex estimation, researchers develop sex-specific criteria using statistical analysis and comparative investigations. PRH is also a useful tool for investigating morphological differences within and between populations in population studies and anthropological research [15]. Researchers can learn more about hereditary factors, environmental adaptations, and evolutionary processes by analyzing PRH data from a variety of groups [16].

BGW measures the horizontal separation between the mandible's two gonial angles. Bigonial breadth is influenced by sexual dimorphism, with males often measuring wider than females. This variation in mandibular shape between the sexes is caused by biomechanical requirements, genetic influences, and hormonal effects [17]. BGW measures, in conjunction with other mandibular metrics, are employed by forensic practitioners and researchers to precisely determine the biological sex of skeletal remains. This information is utilized to support both forensic investigations and anthropological studies [18,19]. 

The GA refers to the angle that is created where the mandibular body and ramus meet. Because of sexual dimorphism, the GA differs between the sexes, with males typically having a more obtuse angle than females [20]. Hormonal impacts, genetic variables, and biomechanical stress are the reasons for this disparity. GA measures, along with other mandibular metrics, are used by forensic specialists and researchers to properly determine the biological sex of skeletal remains. This information is useful for both forensic investigations and anthropological studies [21,22]. 

An important feature in craniofacial anatomy, the AGA, is located where the ramus and mandibular body meet. Analyzing the AGA's size and form in light of gender-specific standards is necessary to determine a person's sex [23]. Research indicates that when compared to females, males often have greater and more obtuse AGA [24,25]. This anatomical distinction helps determine the biological sex of skeletal remains, which facilitates forensic identification [26,27].

For the included studies, study characteristics involving the author's name, published year, sample size, age, type of study, the population included, and mean and standard deviation values obtained are discussed in Table 2.

**Table 2:** Characteristics of the studies included in the systematic review The parameters taken for assessments are PRH, BGW, GA, AGA, MiRB, and MaRB PRH: projective ramus height; BGW: bigonial width; GA: gonial angle; AGA: antegonial angle; MiRB: minimum ramus breadth; MaRB: maximum ramus breadth

S. no.	Author's name	Year of publication	Sample size		Mean age		Type of study	Population	Parameters	Male		Female	
			Male	Female	Male	Female				Mean	SD	Mean	SD
1	Ingaleshwar et al. [7]	2022	100	100	18-58	18-58	Population study	India	GA	120.47	8.4	124.98	6.21
2	Shah et al. [17]	2020	150	150	20-50	20-50	Population study	India	MiRB	28.35	3.01	26.51	3.53
3	More et al. [21]	2017	500	500	21-60	21-60	Population study	India	PRH	68.14	5.65	63.09	5.67
4	Satish et al. [22]	2017	100	100	18-30	18-30	Population study	India	BGW	171.61	10.21	159.8	8.04
5	Dosi et al. [23]	2018	273	272	>20	>20	Retrospective study	India	PRH	47.02	3.6	47.3	3.43
6	Bhuyan et al. [24]	2018	25	25	10-80	10-80	Retrospective study	India	PRH	64.56	1.06	57.16	1.04
									BGW	162.97	2.34	156.49	2.21
									GA	128.43	2.02	117.1	1.83
7	Osato et al. [25]	2012	117	117	20-78	20-78	Population study	Japan	AGA	160.55	7.03	162.18	7.35
8	Ogawa et al. [26]	2012	78	78	49.54	49.54	Population study	Japan	AGA	164.17	3.27	164.47	4.14
9	Ulusoy and Ozkara [27]	2022	550	550	3-13	3-13	Population study	Turkey	MaRB	33.29	3.23	31.25	3.28

Limitations

The limitations of the study include morphometric features that can vary between populations, ethnic groups, and geographical regions. Also, morphology can change with age-related processes such as growth, tooth loss, and bone remodeling. Care must be taken to ensure all these are taken into consideration. To improve the accuracy and reliability of gender estimation using mandibular parameters, more research is required to standardise measuring methods and validate them across a range of populations.

## Conclusions

Out of four mandibular parameters, only BGW is a useful tool in identifying gender, whereas PRH, GA, and AGA cannot be preferred. However, there are a limited number of studies and high heterogeneity among the included studies. Therefore, any general conclusions should be drawn cautiously. As a result, the clinical significance of the discrepancy cannot be confirmed and should be explained with further research.
